# Overcoming Antimicrobial Resistance in Bacteria Using Bioactive Magnetic Nanoparticles and Pulsed Electromagnetic Fields

**DOI:** 10.3389/fmicb.2017.02678

**Published:** 2018-01-09

**Authors:** Vitalij Novickij, Ramunė Stanevičienė, Iglė Vepštaitė-Monstavičė, Rūta Gruškienė, Tatjana Krivorotova, Jolanta Sereikaitė, Jurij Novickij, Elena Servienė

**Affiliations:** ^1^Institute of High Magnetic Fields, Vilnius Gediminas Technical University, Vilnius, Lithuania; ^2^Laboratory of Genetics, Institute of Botany, Nature Research Centre, Vilnius, Lithuania; ^3^Department of Chemistry and Bioengineering, Vilnius Gediminas Technical University, Vilnius, Lithuania; ^4^Institute of Chemistry, Vilnius University, Vilnius, Lithuania

**Keywords:** antimicrobial resistance, bacteria inactivation, *B. subtilis*, *E. coli*, iron oxide nanoparticles, nisin

## Abstract

Nisin is a known bacteriocin, which exhibits a wide spectrum of antimicrobial activity, while commonly being inefficient against Gram-negative bacteria. In this work, we present a proof of concept of novel antimicrobial methodology using targeted magnetic nisin-loaded nano-carriers [iron oxide nanoparticles (NPs) (11–13 nm) capped with citric, ascorbic, and gallic acids], which are activated by high pulsed electric and electromagnetic fields allowing to overcome the nisin-resistance of bacteria. As a cell model the Gram-positive bacteria *Bacillus subtilis* and Gram-negative *Escherichia coli* were used. We have applied 10 and 30 kV cm^-1^ electric field pulses (100 μs × 8) separately and in combination with two pulsed magnetic field protocols: (1) high d*B*/d*t* 3.3 T × 50 and (2) 10 mT, 100 kHz, 2 min protocol to induce additional permeabilization and local magnetic hyperthermia. We have shown that the high d*B*/d*t* pulsed magnetic fields increase the antimicrobial efficiency of nisin NPs similar to electroporation or magnetic hyperthermia methods and a synergistic treatment is also possible. The results of our work are promising for the development of new methods for treatment of the drug-resistant foodborne pathogens to minimize the risks of invasive infections.

## Introduction

Consumption of food, which is contaminated by pathogenic bacteria represents a serious public health problem (Pinilla and Brandelli, 2016). The drug-resistant foodborne pathogens are of greatest concern due to the invasive infections, high risks of death, and massive outbreaks of disease (Roca et al., 2015; Lammie and Hughes, 2016). The situation is complicated by the increasing rates of antimicrobial resistance, which can ultimately be a consequence of the abuse or misuse of antibacterial agents (Roca et al., 2015). Therefore, the applied research of antimicrobials, alternative or combinational methods for the biocontrol, and sensitization of pathogenic microorganisms are in constant focus (Ristic et al., 2014; Ziuzina et al., 2015; Campion et al., 2017). The main interest lies within the development of natural additives and minimally processed foods to preserve taste and nutritional value (Chemat et al., 2017). Nevertheless, the array of available food preservatives, generally recognized as safe (GRAS) and approved by EU and US food and drug administration committees is limited (Gharsallaoui et al., 2016).

One of such prominent bacteriocins (E234) unspotted in health problems is nisin, an oligopeptide produced by certain strains of *Lactococcus* (Cotter et al., 2012; Rao et al., 2016). Nisin is a cationic peptide composed of 34 amino acid residues. It belongs to lantibiotics and contains unusual amino acid residues dehydrobutyrine, dehydroalanine, lanthionine, and β-methyl lanthionine. The molecular mass of nisin is 3.5 kDa. However, at higher pH values (8–9) nisin is prone to form multimers (Gharsallaoui et al., 2016). It is active against a broad spectrum of Gram-positive foodborne pathogens; however, its use as a biopreservative is limited due to low efficiency against Gram-negative bacteria (Gharsallaoui et al., 2016; Pinilla and Brandelli, 2016). Moreover, the repeated exposure of bacteria to increasing nisin concentrations leads to patterns of nisin resistance, which also participate in the resistance to other antimicrobials or antibiotics (Blake et al., 2011; Zhou et al., 2014). Gram-negative bacteria are resistant to nisin mainly due to impermeable outer membranes, since nisin has to incorporate itself in the bacterial cell membrane by binding to essential precursors for cell wall biosynthesis, which ultimately leads to formation of pores, loss of solutes in bacteria, and subsequent cell death (Wiedemann et al., 2004; Campion et al., 2017).

The straightforward solution is to use combinational methods to improve the bacteria inactivation efficiency (i.e., garlic extracts, CO_2_, pressure, thermal stress), which all showed an improvement in nisin-mediated biopreservation (Lee and Kaletunç, 2010; Al-Holy et al., 2012; Chandrasekaran et al., 2013; Pinilla and Brandelli, 2016; Rao et al., 2016). However, since the bacteriocins can lose their antimicrobial activity due to environmental factors such as pH, temperature, or food composition (Zhou et al., 2014) additional encapsulation and use of nanostructures as carriers are advantageous (Lopes and Brandelli, 2017). Development of nisin-loaded nanoparticles (NPs) can improve the stability and efficiency of the nisin-mediated antimicrobial treatment (Prombutara et al., 2012; Zohri et al., 2013; Krivorotova et al., 2016). However, the physical methods that are used in combination should be targeted and preferably non-thermal to guarantee high stability and efficiency of nisin treatment, preserve nutritional value, and quality of food (Keenan et al., 2010; Rawson et al., 2011). Also, since nisin employs a plasma membrane interaction mechanism and its efficiency is highly dependent on the pore formation, the applied physical methods should be synergistic and exhibit similar effects (Novickij et al., 2016b). The perfect candidate is the pulsed electric field (PEF) processing methodology or electroporation, which triggers reversible or irreversible process of pores formation in the cell wall or cell membrane (Kotnik et al., 2012; Mahnič-Kalamiza et al., 2014; Pillet et al., 2016). The method is non-thermal and already made its way to food science and food processing as a sterilization or extraction method (Bobinaite et al., 2014; Golberg et al., 2016; Pal, 2017). However, electroporation is an emerging technique for sensitization of bacteria to antimicrobials (Khan et al., 2016; Novickij et al., 2016b; Ravensdale et al., 2016). Recently, a proof of concept that it is possible to overcome the nisin-resistance of Gram-negative bacteria using electroporation and nisin-loaded NPs was presented (Novickij et al., 2016b).

As an alternative to electroporation, a pulsed electromagnetic field (PEMF) methodology has been proposed recently (Kardos and Rabussay, 2012; Towhidi et al., 2012; Novickij et al., 2017a). The concept is based on generation of the time-varying pulsed magnetic field, which induces electric field and triggers contactless electroporation (Kranjc et al., 2016). The PEMF method has an additive effect with PEF, which allows significant increase of the treatment efficiency without contamination (Novickij et al., 2016a, 2017c). It is a fundamentally new phenomenon, which can be triggered only in extremely high magnetic fields, while the exact mechanism of the effect currently is not fully understood. However, weak PEMF-based methodologies are well studied and are frequently used in combination with magnetic NPs (Lee et al., 2011; Chandrasekaran et al., 2013; Vallejo-Fernandez et al., 2013; Niemirowicz et al., 2016). Magnetic NPs have attracted increasing attention as an efficient tool in various areas of application. In the biomedical field, they are used for magnetic hyperthermia, thermoablation therapies, targeted drug delivery, and as contrast agents for magnetic resonance imaging (Wu et al., 2015). In biotechnology, iron oxide magnetic NPs functionalized with various biomolecules find their application in the biological separation, biosensing, bioremediation, and magnetofection process (Assa et al., 2016; Dinali et al., 2017).

Therefore, we have speculated that it is possible to increase the antimicrobial efficiency of nisin using different encapsulation to improve stability and binding of the structure to magnetic nano-carriers, while the nisin-resistance can be overcome by controlled poration of the cell membrane in PEF. The PEMF methodology can be further used to increase the treatment efficiency. As a result, we are first to present a proof of concept of novel antimicrobial methodology using targeted magnetic nisin nano-carriers, which are activated by combination of electric and high PEMF.

## Materials and Methods

### Pulsed Power Methods

The experimental setup consisted of three generators: (1) up to 3 kV, 100 ns – 1 ms square wave high voltage pulse generator; (2) up to 3.3 T, high d*B*/d*t* generator, where *B* – magnetic flux density; and (3) 10 mT, 100 kHz PEMF generation setup. The first experimental setup generated electrical pulses (1 and 3 kV) in a sequence of eight pulses (1 Hz) in a commercially available 1 mm gap electroporation cuvette (Biorad, Hercules, CA, United States). A fixed duration of 100 μs was used, which is typical in electroporation studies (Haberl Meglic et al., 2015). The resultant electric field in the cuvette was 10 and 30 kV/cm, respectively.

We have used two pulsed magnetic field setups to induce different magnetic phenomena. The high d*B*/d*t* generator was based on Marx circuit topology. The total discharge voltage was 23 kV, resulting in up to 5 kA current in the coil (two layers, six windings), which was compatible (inner diameter of 5.2 mm) with 0.2 ml polymerase chain reaction (PCR) sterile tubes (Quali Electronics Inc., Columbia, SC, United States). A maximum amplitude of 3.3 T could be generated.

**FIGURE 1:**
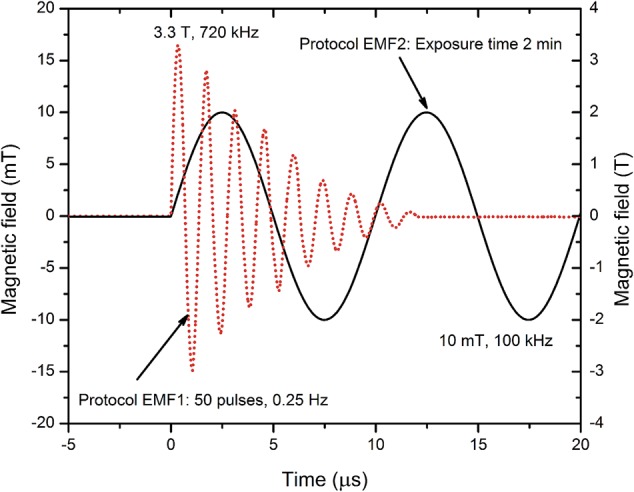
Applied magnetic field protocols. The pulses have been measured using a calibrated loop sensor (VGTU, Vilnius, Lithuania), a DPO4034 oscilloscope (Tektronix, Beaverton, OR, United States), and a Gaussmeter 475DSP (Lakeshore, Carson, CA, United States), post-processed in OriginPro Software (OriginLab, Northampton, MA, United States).

The second PEMF setup was based on the high frequency resonance oscillator with maximum current support of 70 A. As a load 25 mm inner diameter coil was used (single layer, eight windings), which was compatible with standard 1.5 ml Eppendorf tubes (Eppendorf, Hamburg, Germany). A maximum amplitude of 10 mT could be generated at a 100 kHz frequency. The measured waveforms of both PEMF generators and the protocols for treatment of the cells are presented in **Figure 1**.

The protocol EMF1 was for the high d*B*/d*t* system and included 50 oscillations generated at repetition frequency of 0.25 Hz (total treatment time of 3 min 20 s). High d*B*/d*t* component allowed to induce electric field of 0.2 kV cm^-1^ inside the sample and thus potentially enable interaction with the plasma membrane of the cell to promote permeabilization.

The protocol EMF2 was developed for the high frequency PEMF setup and was dedicated for the induction of local hyperthermia and additional motion of NPs inside the sample. The cells were subjected to 10 mT, 100 kHz PEMF for 2 min. In both cases, the methodologies were contactless; however, the concomitant delivery of magnetic field pulses after electric field pulses was used to determine if a phenomenon of increased inactivation efficiency by PEF and PEMF can be triggered (Novickij et al., 2016a).

### Preparation of Nanoparticles

#### Materials

FeSO_4_.7H_2_O (≥99% p.a.), α(+)-ascorbic acid (≥99% p.a.) (Asc), and gallic acid (Gal) monohydrate were purchased from Roth, citric acid (Ca) monohydrate, and FeCl_3_.6H_2_O were obtained from Fisher Chemicals and Merck, respectively. Nisin (NisinZ^TM^ P) was purchased from Handary S.A. (Brussels, Belgium). All materials were used without additional purification.

#### Preparation of Nisin-Loaded Iron Oxide Magnetic Nanoparticles

Nisin-loaded iron oxide magnetic NPs were prepared as previously described (Gruskiene et al., 2017). Briefly, under vigorous stirring, 0.587 g FeC_3_.6H_2_O and 0.278 g FeSO_4_.7H_2_O were mixed in 10 ml of water and heated to 80°C under nitrogen in a three-necked flask. Then, 3.5 ml of NH_4_OH (10%) was dropped into the solution. After reaction for 30 min at 80°C, 0.3 g of Ca (Asc or Gal) in 0.6 ml water was added directly into the reaction solution. The temperature was increased to 95°C, and stirring continued for an additional 90 min. Then, the solution was cooled down to room temperature naturally. The iron oxide particles were separated by a magnet from reaction mixture, washed with deionized water for several times, and dried at 45°C for 12 h. The prepared dried powder was stored in the refrigerator. Before using, the required amount of iron oxide was redissolved in water using an ultrasonic water bath for 3 h and centrifuged at 6400 × g for 2 h. The final iron oxide nanoparticles (IONP) solution was used for the following nisin loading. Synthesized iron oxide nanoparticles capped with Ca (IONP-Ca), Asc (IONP-Asc), or Gal acid (IONP-Gal) corresponded to Fe_2_O_3_ phase (Maghemite-C, ICDD Card No. 00-039-1346) as judged by X-ray diffraction method.

For the preparation of nisin-loaded particles, a volume of nisin solution in water at the concentration of 10 μg/ml was added dropwise to the IONP solution (0.05 mg/ml) at the ratio 1/4 (v/v) under constant stirring at room temperature. For the preparation of control, instead of nisin solution, water was used. The solution of prepared nisin loaded IONP was stored at +4°C. Nisin loading on the particles was confirmed by Fourier transform infrared spectroscopy and thermogravimetric analysis.

The average diameter of nisin-loaded IONP-Ca, IONP-Asc, and IONP-Gal was equal to 11, 13, and 12 nm, respectively, as determined by atomic force microscopy. Nisin-loaded NPs were stable at least for 6 weeks as judged by dynamic light scattering method. The concentration of nisin for all NPs was 2 μg/ml.

### Bacterial Cultures and Growth Conditions

Bacteria representing both Gram-positive and Gram-negative microorganisms were selected in accordance with widely adopted model organisms for laboratory studies and industrial application of PEF and food technology (Pillet et al., 2016; Murashita et al., 2017; Pan et al., 2017). Gram-negative bacteria *Escherichia coli* BL21 [F-dcm ompT hsdS(rB-mB-) gal (DE3)] (ThermoFisher Scientific, Vilnius, Lithuania) and Gram-positive bacteria *Bacillus subtilis* ATCC 6633 (kindly provided by the Vilnius University, Vilnius, Lithuania) were propagated in Luria-Bertani (LB) medium (2% tryptone, 2% yeast extract, 1% NaCl) for 16–18 h with continuous shaking at 37°C. For exponential growth, overnight cultures were transferred to fresh LB medium and incubated at 37°C for additional 3 h.

### Analysis of Antimicrobial Activity by Agar Plate Count Method

Stationary and exponential *B. subtilis* and *E. coli* cells (1 × 10^7^–1 × 10^8^ cells/sample) were collected by centrifugation at 6000 × *g* for 5 min, washed with 0.9% NaCl solution, suspended in 100 μl of solution containing nisin unloaded/loaded IONP, and used for electric and/or high PEMF treatment. Afterward samples were incubated at room temperature (20°C) for 2 h, serial dilutions performed in 0.9% NaCl, and 50 μl of each solution was spread onto LB-agar plates with following incubation overnight at 37°C. All assays were carried out in triplicate. After incubation, colonies were counted as colony forming units (CFU), and then the mean value of CFU/ml was calculated.

### Statistical Analysis

One-way analysis of variance (ANOVA; *P* < 0.05) was used to compare different protocols. Tukey’s HSD multiple comparison test for evaluation of the difference was used when ANOVA indicated a statistically significant result (*P* < 0.05 was considered statistically significant). The data were post-processed in OriginPro Software (OriginLab, Northampton, MA, United States). All experiments have been performed at least in triplicate and the treatment efficiency was expressed as mean ± standard deviation normalized to untreated control sample.

## Results

The antimicrobial activity of NPs depends on the encapsulation method used, and therefore, we have used IONP with different capping agents. Citric and Asc are well-known stabilizers of NPs (Moore et al., 2015; Sreeja et al., 2015). Recently, Gal has been used for coating of IONP, which are applicable for trypsin immobilization by physical bonds (Atacan et al., 2016). However, these acids differ in their chemical structure and properties and consequently may influence on the chemisorption of nisin and its biological activity. Therefore, each nisin-loaded version of NPs (i.e., Nis-IONP-Ca) had a corresponding nisin-free version as a reference (IONP-Ca) to distinguish the influence of separate treatment components. The NPs have been used in combination with PEMF to induce higher inactivation efficiency.

### Inactivation in Pulsed Electric Fields

**FIGURE 2:**
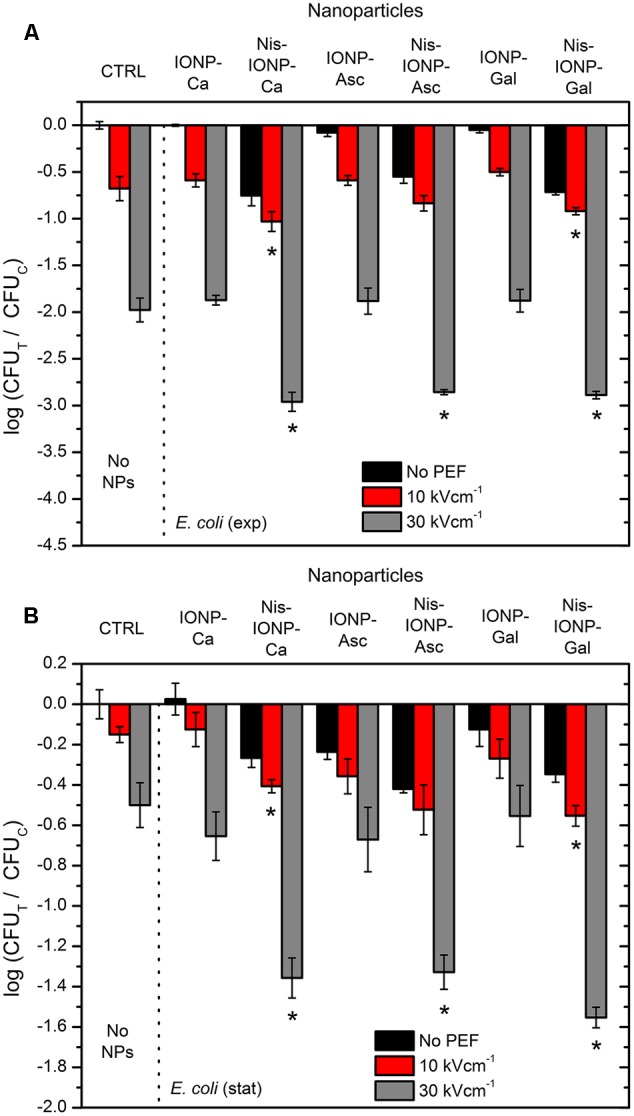
Inactivation of *Escherichia coli* using different pulsed electric field (PEF) protocols and nanoparticles (NP), where **(A)**, exponential bacteria growth phase; **(B)**, stationary bacteria growth phase. CTRL, control samples without NPs; IONP, iron oxide nanoparticles; Nis, nisin-loaded. Different capping agents were used such as citric (Ca), ascorbic (Asc), and gallic (Gal) acids. The number of residual culturable cells in the samples after the PEF treatment (CFU_T_) was compared with ones in the control samples without treatment (CFU_C_). The asterisk (^∗^) represents statistically significant (*P* < 0.05) difference versus untreated control.

The conventional protocol of 8 × 100 μs was used with the bacteria during the exponential and the stationary growing phase. The results for *E. coli* are summarized in **Figure 2**. Both the nisin-loaded and nisin-free NPs did not result in high inactivation rates (up to 0.75 log CFU reduction) when used separately from electroporation. However, 30 kV cm^-1^ pulsing protocol showed a high increase in antimicrobial efficiency of the treatment (up to 3 log CFU reduction).

When PEF was used separately from NPs, up to 2 log reduction in cell survival was detected (**Figure 2A**, 30 kV cm^-1^), while in all cases with the nisin-loaded NPs a synergistic treatment was triggered. The 10 kV cm^-1^ PEF procedure showed a less profound effect (up to 1 log reduction); however, still in Nis-IONP-Ca and Nis-IONP-Gal cases the difference was significant (**Figure 2A**, 10 kV cm^-1^). The same tendency was observed for *E. coli* in the stationary growth phase. Bacteria were less susceptible to treatment resulting in a maximum 1.6 log reduction in cell survival (PEF + NPs), nevertheless a significant improvement in inactivation efficiency was detectable if compared to separate procedures.

**FIGURE 3:**
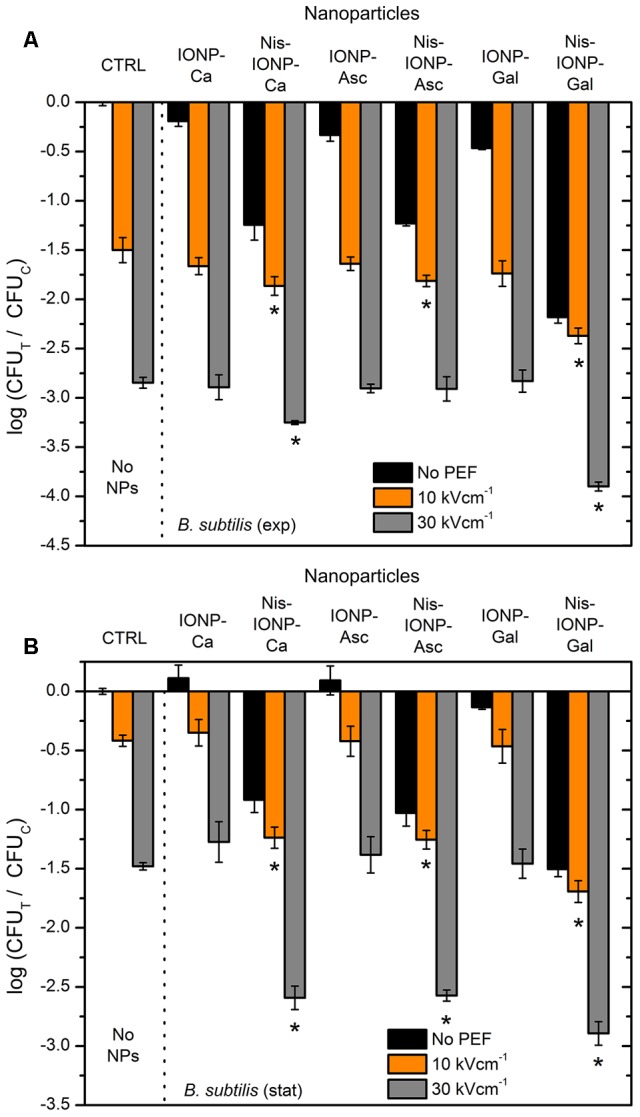
Inactivation of *Bacillus subtilis* using different PEF protocols and NP, where **(A)**, exponential bacteria growth phase; **(B)**, stationary bacteria growth phase. CTRL, control samples without NPs; IONP, iron oxide nanoparticles; Nis, nisin-loaded. Different capping agents were used such as Ca, Asc, and Gal acids. The number of residual culturable cells in the samples after the PEF treatment (CFU_T_) was compared with ones in the control samples without treatment (CFU_C_). The asterisk (^∗^) represents statistically significant (*P* < 0.05) difference versus untreated control.

The same methodology was applied for Gram-positive bacteria (i.e., *B. subtilis*). The results are summarized in **Figure 3**. More than 2 log reduction in cell survival was triggered solely by nisin-loaded NPs (**Figure 3A**, Nis-IONP-Gal). Also, the electroporation protocols were more effective, however, the difference between combinational (PEF + NPs) and PEF only treatment was not as apparent as in *E. coli* case for most of the used NPs, which indicates a saturated permeabilization.

The results for *B. subtilis* in stationary growth phase agree with previously observed phenomena for *E. coli*. The synergistic treatment efficiency was detectable in all cases when nisin-loaded NPs were used in combination with PEF (**Figure 3B**).

### Inactivation in Pulsed Electromagnetic Fields

**FIGURE 4:**
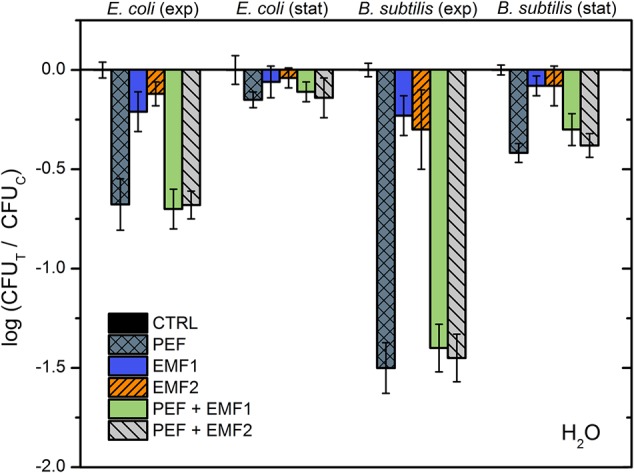
Inactivation of *E. coli* and *B. subtilis* using different treatment protocols without NP, where PEF – 10 kV cm^-1^ × 8 × 100 μs; EMF1 – 3.3 T × 50, 0.25 Hz; EMF2 – 10 mT, 100 kHz, 2 min. The number of residual culturable cells in the samples after the PEF treatment (CFU_T_) was compared with ones in the control samples without treatment (CFU_C_).

Firstly, we have investigated the influence of EMF protocols (with and without PEF component) in distilled water. The 10 kV cm^-1^ protocol was selected on purpose to prevent occurrence of saturated permeabilization and thus, potentially enable detection of synergistic response. The results for both bacteria are summarized in **Figure 4**.

**FIGURE 5:**
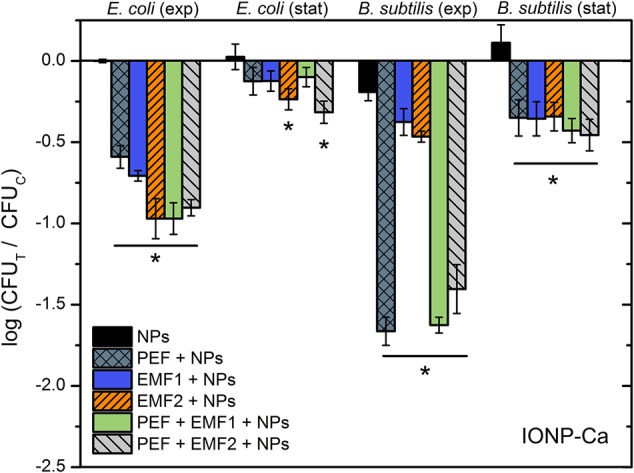
Inactivation of *E. coli* and *B. subtilis* using different treatment protocols and nisin-unloaded iron oxide NP with Ca as a capping agent (IONP-Ca), where PEF – 10 kV cm^-1^ × 8 × 100 μs; EMF1 – 3.3 T × 50, 0.25 Hz; EMF2 – 10 mT, 100 kHz, 2 min. The number of residual culturable cells in the samples after the PEF treatment (CFU_T_) was compared with ones in the control samples without treatment (CFU_C_). The asterisk (^∗^) represents statistically significant (*P* < 0.05) difference versus NPs only treatment.

As it can be seen in **Figure 4**, both the EMF protocols are barely influencing the survival of bacteria. Also, no additive effect with PEF was detected. Considering the acquired data, we have added NPs to include additional interactions of electromagnetic field. However, based on the inactivation efficiencies in PEF, we have narrowed the NPs to a pair of nisin-loaded (Nis-IONP-Ca) and nisin-free versions (IONP-Ca), which showed a stable antimicrobial response for both bacteria previously (**Figures 2, 3**). Then, the bacteria suspended with the selected NPs were treated in accordance with the methodology described above. The results for IONP-Ca and both bacteria are presented in **Figure 5**.

As it can be seen in **Figure 5**, the *E. coli* during exponential growth phase reacted to all of the applied external stimuli with both EMF protocols being similarly effective or even better than the 10 kV cm^-1^ PEF (up to 1 log reduction). The result was not so apparent for the *B. subtilis*, nevertheless both the EMF protocols showed an increase in antimicrobial efficiency if compared to NP-treatment only. Bacteria in stationary growth phase showed a weak response to the treatment, which is in agreement with the experimental data in PEF (**Figures 2, 3**); however, the synergistic response for the EMF2 + PEF protocol was still detectable for *E. coli*.

**FIGURE 6:**
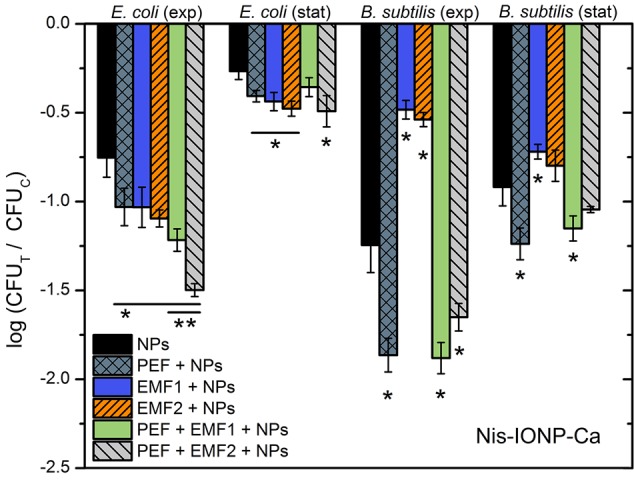
Inactivation of *E. coli* and *B. subtilis* using different treatment protocols and nisin-loaded iron oxide NP with Ca as a capping agent (Nis-IONP-Ca), where PEF – 10 kV cm^-1^ × 8 × 100 μs; EMF1 – 3.3 T × 50, 0.25 Hz; EMF2 – 10 mT, 100 kHz, 2 min. The number of residual culturable cells in the samples after the PEF treatment (CFU_T_) was compared with ones in the control samples without treatment (CFU_C_). The asterisk (^∗^) represents statistically significant (*P* < 0.05) difference versus NPs only treatment. The asterisk (^∗∗^) represents statistically significant (*P* < 0.05) difference versus PEF + NPs treatment.

Results for the nisin-loaded version of NPs (Nis-IONP-Ca) are summarized in **Figure 6**. As it can be seen in **Figure 6**, a similar tendency is apparent for *E. coli*. A clear increase in inactivation efficiency is observed when the synergistic EMF + PEF protocols were applied. However, we have detected a significant reduction of treatment efficiency for *B. subtilis* when the NPs were used with EMF protocols. The effect was also apparent during combinational protocol (EMF2 + PEF).

## Discussion

Various emerging technologies for food processing are introduced every year with a hope to present an economical, effective, and simple methodology for successful biopreservation of food without reduction of food quality. In this work, we have presented a novel method using targeted magnetic nisin nano-carriers, which are activated by combination of electric and high PEMF, allowing to overcome the initial nisin-resistance of *E. coli*. We have developed magnetic IONP and increased the antimicrobial efficiency of treatment using pulsed electric and magnetic fields. As a result, a proof of concept has been presented that the high pulsed magnetic fields increase the antimicrobial efficiency of nisin NPs similar to electroporation or magnetic hyperthermia methods, while a synergistic treatment is also possible.

We have shown that the electroporation can be effective by itself (30 kV cm^-1^) with or without the nisin NPs, which is in agreement with other PEF works (Žgalin et al., 2012; Flisar et al., 2014; Pataro et al., 2014). The effect could be attributed to the high permeabilization, which is induced due the increase of the transmembrane voltage in PEF (Zou et al., 2015). The cell wall of bacteria serves as an ultimate barrier against environment, however, electric field higher than 10 kV cm^-1^ is already sufficient to cause irreversible cell wall deterioration induced by both the mechanical and physical damage (Pillet et al., 2016). The plasma membrane protects Gram-negative bacteria from nisin incorporation, however, the application of PEF allows to permeabilize the bacteria and thus sensitize them to nisin treatment, which is in agreement with the currently known mechanisms of nisin resistance and PEF effects (Wouters et al., 2001; Vesković Moračanin et al., 2014; Zhou et al., 2014; Gharsallaoui et al., 2016). However, the combination of nisin NPs with PEMF was of upmost interest.

The high d*B*/d*t* protocol proved to be as effective as the 10 kV cm^-1^ PEF procedure for the *E. coli*. Nevertheless, without the NPs both EMF protocols were ineffective. The explanation could lie within the mechanism behind the high pulsed magnetic field treatment. The high d*B*/d*t* treatment is an emerging technique, however, the dominant idea is that the high magnetic field can result in induction of additional transmembrane potential in the cell and thus, stimulate permeabilization or affect the activity of ion channels (Towhidi et al., 2012; Kranjc et al., 2016; Zablotskii et al., 2016; Polyakova et al., 2017). The inability to affect the survival of bacteria solely by EMF is an expected result, due to relatively low (up to 0.2 kV cm^-1^) induced electric field if compared to the 10 kV cm^-1^ PEF treatment. The result is in agreement with the established electroporation theory, indicating that higher d*B*/d*t* magnetic fields are required to permeabilize bacteria (Chen et al., 2006; Towhidi et al., 2012; Kranjc et al., 2016; Rems and Miklavčič, 2016; Novickij et al., 2017b). On contrary, magnetic NPs in combination with EMF induce significantly higher local field gradients, hyperthermia, and motion of both the cells and NPs, which all could attribute to increased inactivation efficiency (Deatsch and Evans, 2014; Salunkhe et al., 2014; Polyakova et al., 2017). The effect is apparent both with nisin-loaded and nisin-free NPs, which confirms the hypothesis.

Lastly, we have shown that both EMF protocols result in additive effects with PEF for inactivation of *E. coli* using nisin-loaded NPs, however, a significant reduction in treatment efficiency was observed for *B. subtilis*. *B. subtilis* is initially permeable to nisin, while high PEF exposure results in saturated permeabilization, which can explain the absence of additive effect with EMF. However, the decreased inactivation efficiency could be the cause of dielectrophoretic movement of cells and electromotive forces that are induced during EMF exposure, affecting passive diffusion and incorporation of nisin in the membrane. Currently, it is not possible to determine the exact mechanism and this phenomenon requires further investigation in future works.

We conclude that EMF is a versatile tool, which can be successfully used both separately and in combination with electroporation for sensitization of bacteria to antimicrobial peptides. However, further research is required in order to better understand the mechanisms of effect, determine the optimal parameters, and optimize the treatment efficiency. From the technological point of view, EMF methods are advantageous due to ease of incorporation in existing food processing systems, contactless treatment possibility, and thus absence of electrolysis and contamination, while we have shown a proof of concept that additive effects with PEF are also possible.

## Author Contributions

VN, ES, and JS conceived the experiments and methodology. RG, TK, and JS developed and produced the NPs, and performed the characterization. VN, RS, IV-M, and ES conducted the experiments, processed, and analyzed the results. VN and JN developed the pulsed magnetic and electric field systems. VN, ES, JS, and JN interpreted the results and wrote the manuscript. All authors reviewed and approved the final manuscript.

## Conflict of Interest Statement

The authors declare that the research was conducted in the absence of any commercial or financial relationships that could be construed as a potential conflict of interest.
